# Effect of Yin-Xing-Tong-Zhi Tablets on Improving Vascular Cognitive Impairment No Dementia

**DOI:** 10.1155/2018/6184260

**Published:** 2018-04-26

**Authors:** Weidong Pan, Wei Zhu, Qi Gao, Weilong Liao, Penglin Gao, Te Liu

**Affiliations:** ^1^Department of Neurology, Shuguang Hospital Affiliated to Shanghai University of Traditional Chinese Medicine, Shanghai, China; ^2^Department of Neurology, Gongli Hospital, The Second Military Medical University, Shanghai, China; ^3^SPH Xingling Technology Pharmaceutical Co., Ltd., Shanghai, China; ^4^Shanghai Geriatric Institute of Chinese Medicine, Longhua Hospital, Shanghai University of Traditional Chinese Medicine, Shanghai, China

## Abstract

**Objective:**

Observe the effect of the Chinese herbal extracts, Yin-Xing-Tong-Zhi, in tablet form, on improving vascular cognitive impairment no dementia (VCIND).

**Methods:**

Sixty-eight patients with VCIND were divided randomly into treatment and placebo groups with oral administration of Yin-Xing-Tong-Zhi tablets (YXTZTs) or placebo, respectively, for 24 weeks. Alzheimer's Disease Assessment Scale-Cognitive (ADAS-Cog) subscale score, MMSE score, Clinician's Interview-Based Impression of Change Plus Caregiver Input (CIBIC-Plus) score, expression of interleukin- (IL-) 6, IL-8, and tumor necrosis factor- (TNF-) *α* in serum, and variation of blood-lipid levels were evaluated at different time points.

**Results:**

At weeks 12 and 24, the scores for the ADAS-Cog, CIBIC-Plus, and MMSE of the treatment group were significantly lower than those of the control group (*P* < 0.05). All clinical scales at week 24 of the control group were significantly different from those before treatment (*P* < 0.05). Expression of IL-6, IL-8, and TNF-*α* in the two groups was reduced significantly with variation of the clinical scales of cognitive impairment.

**Conclusion:**

YXTZTs may delay the development of cognitive impairment in VCIND patients by modulating expression of VCIND-associated proinflammatory factors.

## 1. Introduction

Vascular cognitive impairment without dementia (VCIND) is an early stage of vascular dementia (VaD) [1, 2]. The main symptoms can be a decline in understanding, execution, memory, visual spatial skills, and emotions of varying severities and can involve damage to the posterior cingulate cortex and frontal lobe of the brain [3].

It has been accepted that effective treatment could delay the progression of VCIND to VaD [4]. Early studies indicated using special traditional Chinese medicine (TCM) formulas may delay the development or improve the pathologic changes of VaD or AD [5, 6]. Hence, the early diagnosis and treatment of VCIND is very important [7]. Pharmacologic treatment of VCIND is lacking, as are efficacy studies using TCM for VCIND.

Yin-Xing-Tong-Zhi tablets (YXTZTs) are made from the extracts of Yin-Xing-Tong-Zhi, the active ingredients of which are flavone glycosides and terpene lactones [8]. Xiaojian and colleagues showed that YXTZTs can reduce the prevalence of vascular cognitive impairment [9]. However, no scholars have used YXTZTs to prevent or treat cognitive impairment. According to the mechanism of action of YXTZTs in cerebrovascular diseases (CVDs) [10], it can delay VCIND progression and prevent VaD. Here, we conducted a clinical study using YXTZTs in the treatment of VCIND.

## 2. Materials and Methods

### 2.1. Ethical Approval of the Study Protocol

The protocol for our randomized double-blind study was approved by the Ethics Committee of Shuguang Hospital (affiliated to the Shanghai University of Traditional Chinese Medicine, Shanghai, China) and was in accordance with the Declaration of the Helsinki. All patients provided written informed consent to participate in our study.

### 2.2. Diagnostic Criteria

We combined the diagnostic criteria for VCIND formulated by Ingles et al. [4] with the recommendations of Jianping and colleagues for vascular cognitive impairment [11]. The criteria for VCIND were as follows: CVDs or CVD risk factors; fluctuating cognitive impairment; no/mild memory loss; no other severe diseases; causal relationship between CVD and cognitive impairment; ability to carry out the activities of daily living; not meeting the diagnostic criteria for dementia.

### 2.3. Inclusion and Exclusion Criteria

Inclusion criteria were as follows: (i) meeting the diagnostic criteria stated above; (ii) age, 50–70 years; (iii) use of at least one rating scale for clinical dementia, for example, a Mini–Mental State Examination (MMSE) score ≥ 20 for individuals who completed primary education or ≥24 for those who completed secondary or higher education; (iv) magnetic resonance imaging suggesting subcortical ischemic CVD in the brain.

Exclusion criteria were as follows: (i) patients with other diseases that could cause dementia (e.g., multiple sclerosis, cerebral hemorrhage, watershed infarction, atrophy of the hippocampus or olfactory nucleus); (ii) a Hamilton Depression Scale score > 17; (iii) severe neurologic deficits (e.g., aphasia, malaise, and severe hemiplegia); (iv) severe primary diseases of the heart, kidney, endocrine, or hematopoietic systems; (v) mental illness or severe epilepsy; (vi) allergies or allergy to the study drug.

### 2.4. Patient Characteristics

Sixty-eight patients with VCIWD who were inpatients or outpatients from the Department of Neurology of Shuguang Hospital from October 2015 to March 2017 formed the study cohort. Observations ended in August 2017. There were 46 males and 22 females (35–76 years (mean, 52.8 ± 5.9)). The general characteristics of the patients, their clinical-rating scales before study inclusion, and laboratory-related tests are shown in Table 1. All the patients were diagnosed by very experienced neurologists.

### 2.5. Patient Randomization

SPSS v20.0 (IBM, Armonk, NY, USA) was used to generate random-number tables and group numbers at a ratio of 1 : 1 in accordance with the method of complete randomization. Shanghai Xingling Technology Pharmaceuticals were commissioned to produce YXTZTs and placebo according to group numbers. Participants were given the corresponding test drugs according to the order they were included in the clinical observation. The clinical observers and patients did not know the grouping of drugs. Two groups of patients (VCIWD and placebo) were created.

### 2.6. Therapeutic Method

To ensure that patients could manage their comorbidities, they were given the relevant basic treatment. Patients with cerebral infarction were given antiplatelet agents. Cases with hypertension were given calcium antagonists, *β*-blockers, angiotensin-converting enzyme inhibitors, or angiotensin II receptor-blockers. Patients suffering from diabetes mellitus were given insulin secretagogues, biguanides, *α*-glucosidase inhibitors, insulin sensitizers, or insulin. Cases suffering from hyperlipidemia were given statins or fibrates.

Besides the basic treatment stated above, the treatment group were given (at the same time) one YXTZT (t.d.s.). Besides their basic treatment, the control group were given a placebo tablet of the same shape as a YXTZT (t.d.s.). The treatment lasted for 24 weeks. Patients came to a neurology clinic every 2 weeks for further consultation and to obtain YXTZTs/placebos.

### 2.7. Outcome Measures

The primary outcome measures were the Alzheimer's Disease Assessment Scale-Cognitive subscale (ADAS-Cog) score, MMSE score, and the Clinician's Interview-Based Impression of Change Plus Caregiver Input (CIBIC-Plus) score at the day of study inclusion, week 12, and week 24.

The secondary outcome measures were serum levels of interleukin- (IL-) 6, IL-8, and tumor necrosis factor- (TNF-) *α* at the day of study inclusion, week 12, and week 24.

We also measured blood levels of lipids (total cholesterol, high-density lipoprotein, low-density lipoprotein, and triglycerides) and undertook blood biochemical analyses. The urine/stool routine was documented, liver and kidney function noted, and electrocardiography undertaken. These parameters were tested before and after treatment. Also, adverse drug events were monitored.

### 2.8. Statistical Analyses

Data are the mean ± SD. All statistical tests were two-sided. Changes in parameters in the two groups before and after treatment were compared using ANOVA and the Wilcoxon rank sum test, as well as the *χ*^2^ test or nonparametric test. *P* < 0.05 was considered significant. SPSS v20.0 was employed for statistical analyses.

## 3. Results

### 3.1. Demographics

Sixty-eight patients with VCIWD formed the study cohort. Thirty-four patients were in the treatment group and 34 in the placebo group. All 68 patients completed the study.

There were no significant differences in sex, marital status, age, educational level, blood lipids, clinical scale score before treatment, or expression of proinflammatory factors between the two groups (Table 1). Significant liver or kidney dysfunction or significant drug-related adverse events were not observed in either group during treatment.

### 3.2. Primary Outcome Measures

**Figure 1:**
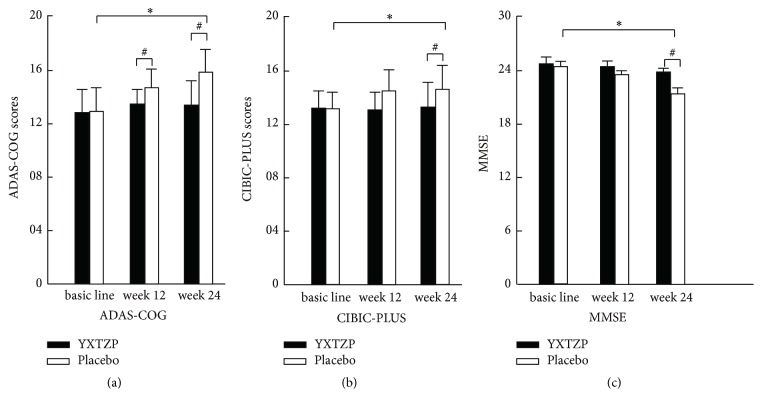
Comparison of clinical scales of the two groups before and after treatment. ^#^*P* < 0.05, comparison between the two groups; ^*∗*^*P* < 0.05, comparison of the same group before and after treatment; YXTZP: Yin-Xing-Tong-Zhi tablets; ADAS-Cog: Alzheimer's Disease Assessment Scale-Cognitive subscale; CIBIC-Plus: Clinician Interview-based Impression of Severity; MMSE: Mini–Mental State Examination.

Compared with the ADAS-Cog scores before treatment, the ADAS-Cog scores at week 12 and week 24 in the treatment group increased, but the differences were not significant (*P* > 0.05). Compared with the ADAS-Cog scores before treatment, the ADAS-Cog scores of the control group increased at week 12 week (*P* > 0.05), and the differences were not significant, but the increased scores at week 24 were not significantly different from those before treatment (*P* < 0.05). At week 24, the ADAS-Cog scores were significantly different between the two groups (*P* < 0.05) (Table 1, Figure 1(a)).

Compared with the CIBIC-Plus scores before treatment, the CIBIC-Plus scores at week 12 and week 24 in the treatment group decreased, but the differences were not significant (*P* > 0.05). However, the CIBIC-Plus scores at week 12 and week 24 in the control group increased, and the differences were not significant compared with those before treatment (*P* > 0.05). The difference value was significantly different between the two groups at week 12 and week 24 (*P* < 0.05) (Figure 1(b)).

Compared with the MMSE scores before treatment, the MMSE scores at week 12 and week 24 in the treatment group decreased, but the differences were not significant (*P* > 0.05). The MMSE score at week 12 in the control group decreased, and the difference was not significantly different compared with that before treatment (*P* > 0.05). Also, the MMSE score at week 24 decreased, and the difference was significantly different compared with that before treatment (*P* < 0.05). The *d* value was significantly different between the two groups at week 24 (*P* < 0.05) (Figure 1).

The data shown above suggested that the cognitive function of the two groups decreased but, at week 24, the cognitive function of the control group decreased more severely.

### 3.3. Secondary Outcome Measures

**Figure 2:**
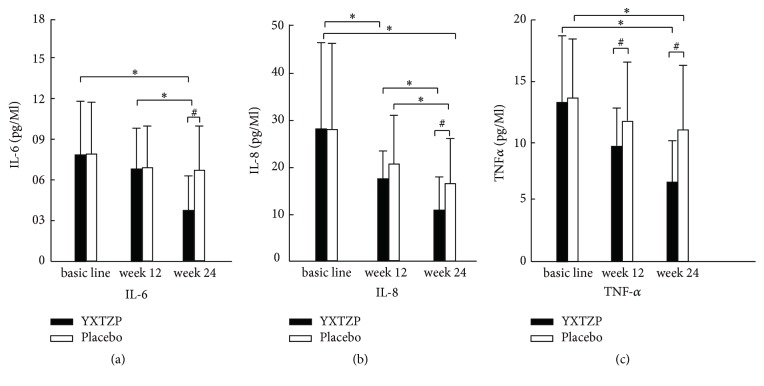
Comparison of changes in levels of proinflammatory factors between the two groups before and after treatment. ^#^*P* < 0.05, comparison between the two groups; ^*∗*^*P* < 0.05, comparison of the same group before and after treatment. YXTZP: Yin-Xing-Tong-Zhi tablets; IL-6: interleukin-6; IL-8: interleukin-8; TNF-*α*: tumor necrosis factor.

In the treatment group, IL-6 expression decreased at week 12 compared with that before treatment, but the difference was not significant (*P* > 0.05). IL-6 expression decreased at week 24, but the difference was significant (*P* < 0.05). In the control group, IL-6 expression decreased at 12 weeks and 24 weeks after treatment, but the difference was not significant (*P* > 0.05). At week 24, the *d* value was significantly different between the two groups (*P* < 0.05) (Figure 2(a)).

In the treatment group, IL-8 expression decreased at week 12 compared with that before treatment, but the difference was not significant (*P* > 0.05). IL-8 expression decreased at week 24, and the difference was significant (*P* < 0.05). In the control group, IL-8 expression increased 12 weeks after treatment, but the difference was not significant (*P* > 0.05). IL-8 expression decreased 24 weeks after treatment, and the difference was significant (*P* < 0.05) (Figure 2(b)).

Compared with TNF-*α* expression before treatment, TNF-*α* expression at week 12 and week 24 in the treatment group decreased significantly, and the difference was significant (*P* < 0.05). In the control group, TNF-*α* expression at week 12 decreased, but the difference was not significant (*P* > 0.05). TNF-*α* expression at week 24 decreased, and the difference was significant (*P* < 0.05) (Figure 2(c)). The *d* value of TNF-*α* between the two groups was significant at week 12 and week 24 (*P* < 0.05).

## 4. Discussion

VCIND is the early stage of VaD and is caused primarily by cerebral arteriosclerosis and ischemic stroke. Hyperlipidemia and the viscosity of cerebral blood flow are important pathogenic factors of cerebral atherosclerosis. VCIND is similar to the pathogenesis of vascular cognitive impairment or VaD [12].

From a TCM perspective, VCIND can be attributed to categories such as “dementia” or “amnesia.” It is a mental disorder with abnormal symptoms caused by physiologic dysfunction, deficiency of essence qi, brain dystrophy, stasis, and other pathogenic factors. The main clinical manifestations are low intelligence and amnesia. Less severe VCIND can result in patients appearing indifferent, less talkative, unresponsive, and forgetful. Combining ancient with modern medical knowledge, we hypothesized that VCIND is an early stage of dementia and, at this stage, drug treatment could help to delay its development.

We conducted a study to ascertain the clinical efficacy of YXTZTs on VCIND. We found that the rate of progression of clinical scales that reflect cognitive function in VCIND patients was delayed significantly after taking YXTZTs for 24 weeks compared with placebos (Figure 1). These differences in efficacy may have involved changes in expression of some proinflammatory factors in atherosclerotic and vascular lesions (Figure 2). Changes in these clinical scales were not related directly to the lipid profiles of VCIND patients.

YXTZTs are used widely in TCM. The preparations within YXTZTs are important for the treatment of CVDs and cardiovascular diseases. These preparations are also used as vegetable supplements in several European countries [13]. TCM theory holds that YXTZ extracts can promote blood circulation to remove blood stasis and can dredge meridians and collaterals. The active ingredients in YXTZTs are flavone glycosides and terpene lactones [14], which can activate the blood circulation to remove blood stasis and open orifices, which in TCM is termed “blood-activating and “orifice-opening.”

Several clinical and basic research studies have shown that YXTZ extracts can improve the symptoms and delay the progression of dementia, improve intelligence levels, and aid the activities of daily living [15–19]. Basic research studies [20] have also suggested that YXTZ extracts can improve the learning and memory of mice with senile dementia and increase the number of hippocampal pyramidal cells in their brain. Studies on VaD have shown good curative effects using YXTZ extracts, and some scholars have speculated that these effects are related to the functions of proinflammatory factors and choline lipase in the brain [21–23]. Hence, YXTZ extracts could aid in helping to delay vascular cognitive impairment.

Studies have shown that expression of proinflammatory markers in the peripheral circulation increases before dementia onset [23]. IL-6 is an important multiple-effect cytokine that has an important role in the immune response. The way that IL-6 affects learning and memory may involve synaptic plasticity and neurogenesis [24]. IL-8 promotes angiogenesis, which has a positive correlation with dementia severity. The mechanism of VaD correlates with increases in the numbers of immune cells and the inflammatory response [25]. TNF-*α* can inhibit neuroinflammatory reactions in rats with VaD and enhance vascular repair to protect the brain [26]. Expression of these proinflammatory cytokines can, to a certain extent, reflect the extent to which the brain suffers cognitive impairment due to vascular factors. Expression of IL-6, IL-8, and TNF-*α* in the present study decreased over time, but it decreased more significantly compared with control patients who took YXTZTs. Cognitive-related symptoms were delayed, but we did not undertake correlation analyses, which was a shortcoming of our study. Another limitation was the small study cohort.

One strength of the present study was that it focused on the early stage of vascular cognitive impairment: VaD. The latter is a nondementia stage during which effective intervention could delay the progression of cognitive impairment. Current thinking is that, irrespective of the type of dementia, once it progresses, it becomes irreversible and will continue to worsen slowly [7, 11]. Our study showed that effective intervention at the early stage of dementia, and treatment for 24 weeks, delayed VCIND progression. Our study showed the slow, stable, and reliable effects of Chinese medicines in the treatment of diseases.

## 5. Conclusions

This clinical study demonstrated that VCIND can be treated and relieved by activating blood and opening orifices. YXTZTs can delay the development of cognitive impairment in VCIWD patients and may alleviate the worsening of VCIND by regulating the associated proinflammatory factors, but there was no significant correlation with blood-lipid profiles.

## Figures and Tables

**Table 1:** Baseline characteristics of the two groups before treatment.

	YXTZTs (*n* = 34)	Placebo (*n* = 34)	*X*2 or *t* or *Z*	*P*
Male/female	20/14	26/8	2.419	0.120
Age	61.76 ± 5.33	62.69 ± 4.32	0.450	0.654
Education (M/H/C)	3/28/3	4/29/1	3.212	0.201
Baseline of ADAS-COG	12.74 ± 2.48	12.76 ± 2.71	0.540	0.589
Baseline of CIBIC-PLUS	13.09 ± 2.56	13.15 ± 1.65	1.778	0.075
Baseline of MMSE	24.00 (23.00, 25.00)	24.00 (24.00, 25.00)	−0.409	0.682
Baseline of IL-6	4.81 (2.54, 14.60)	6.00 (4.09, 12.00)	−0.933	0.351
Baseline of IL-8	25.70 (11.28, 39.13)	18.00 (12.23, 35.20)	−0.276	0.783
Baseline of TNF-*α*	12.70 (8.28, 17.98)	14.00 (12.00, 15.23)	−1.086	1.277

*Note*. YXTZTs: Yin-Xing-Tong-Zhi tablets; M: middle school; H: high school; C: college; ADAS-Cog: Alzheimer's Disease Assessment Scale-Cognitive subscale; CIBIC-Plus: Clinician Interview based Impression of Severity; MMSE: Mini–Mental State Examination; IL-6: interleukin-6; IL-8: interleukin-8; TNF-*α*: tumor necrosis factor.
